# Evaluating the Cytotoxic Effect of Melatonin and Oxyresveratrol on Dental Pulp Stem Cells

**DOI:** 10.5152/eurasianjmed.2023.21270

**Published:** 2023-02-01

**Authors:** Şeyma Keskin, Fatih Şengül, Büşra Şirin

**Affiliations:** 1 Department of Pedodontics, Atatürk University Faculty of Dentistry, Erzurum, Turkey; 2 Department of Pharmacology, Atatürk University Faculty of Medicine, Erzurum, Turkey

**Keywords:** Cell survival, inhibitor concentration 50, melatonin, mesenchymal stem cell, oxyresveratrol

## Abstract

**Objective:**

Hypoxia in the pulpitis process, the use of bleaching agents, and resin-containing materials produce reactive oxygen species. Their damage to the pulp tissue can be eliminated by melatonin and oxyresveratrol. However, the cytotoxic effects of these antioxidants on dental pulp stem cells are not well known. The aim of this study was to observe the cytotoxic effects of melatonin and oxyresveratrol for 72 hours on dental pulp stem cells.

**Materials and Methods:**

Human dental pulp stem cells obtained from the American Type Culture Collection were seeded on E-Plates^®^, and after 24 hours, 3 different doses of melatonin (100 pM, 100 nM, and 100 µM) and oxyresveratrol (10 µM, 25 µM, and 50 µM) were added. Real-time cell index data was acquired by using xCELLigence® device for 72 hours and inhibitor concentration (IC_50_) values of experimental groups were obtained. Analysis of covariance was used to compare the cell index values.

**Results:**

In comparison with the control group; oxyresveratrol 10 µM and melatonin 100 pM groups increased proliferation whereas oxyresveratrol 25 µM, oxyresveratrol 50 µM, and melatonin 100 µM groups induced cytotoxicity (*P *< .05). IC_50_ values at 24th, 48th, and 72th hours were 946 nM, 1220 nM, and 1243 nM for melatonin and 23 µM, 22.2 µM, and 22.5 µM for oxyresveratrol, respectively.

**Conclusion:**

Cytotoxicity of melatonin was higher than oxyresveratrol and they both increased dental pulp stem cells’ proliferation at lower doses and induced cytotoxicity at higher doses.

**Main Points**

- Oxyresveratrol with 10 μM concentration showed maximum dental pulp stem cells (DPSCs) proliferation.
- Melatonin with 100 pM concentration showed maximum DPSCs proliferation.
- Oxyresveratrol was more successful than melatonin in DPSCs proliferation rates.

## Introduction

In the oral cavity, leakage of metal ions from metallic restorations, monomers released from acrylic restorations, hypoxia during the pulpitis process, the use of bleaching agents, and blue light irradiation produce negative effects on pulp and oral cells by generating reactive oxygen species (ROS).^1-5^ Reactive oxygen species, also known as free radicals, may lead to pathologies such as changes in cell function, development of neoplasm, and cell death by damaging the cellular molecules like lipids, proteins, carbohydrates, vitamins, amino acids, and nucleic acids through an oxidation reaction.^6^ Antioxidants that have a major role in ROS detoxification can be used to prevent ROS formation and cellular damage.^7^

Melatonin, a potent endogenous hydroxyl radical scavenger, is involved in several biological functions, especially in the regulation of the circadian rhythm.^8^ Melatonin not only has a wide range of biological effects such as antioxidant, anti-inflammatory, antimitotic, anti-aging, and anti-tumor activity but also has been shown to enhance differentiation in human osteoblasts in vitro and promote bone formation in mice in vivo.^9-11^ It is also thought to have a role in teeth development.^12^ In dental studies, melatonin has shown successful results in improving the post-extraction site wound healing, alleviation of inflammation due to periodontitis and gingivitis, reducing the progressive loss of alveolar bone, treatment of viral infections (e.g., herpes), increasing osteointegration around implants, and management of oral cancers.^9,13^

Oxyresveratrol is an analog of resveratrol (an exogenous antioxidant) with antioxidant, anti-inflammatory, anticancer, antidiabetic, and antiviral activity.^14-18^ In the field of dentistry, oxyresveratrol has positive effects against bacterial and fungal infections and helps in the inhibition and eradication of biofilm formation.^15,19-22^

The idea of adding antioxidants to filling materials has been discussed before in the literature. For example, the effect of N-acetyl cysteine (NAC) addition to resin-modified glass ionomer on rat dental pulp cells was tested. The mineralization capability of rat dental pulp cells was significantly increased by NAC’s antioxidant properties.^23^ Similarly, antioxidants such as melatonin and oxyresveratrol might be used in the field of dentistry by adding them to pulp capping agents, root canal filling pastes, fillers, and bleaching agents to reduce their cytotoxicity.^10,14^ However, before applying these ideas into practice, the biocompatibility of these 2 antioxidants with dental pulp stem cells (DPSCs) must be determined.

The recently developed xCELLigence^®^ system has been used for the real-time monitoring of cell proliferation, viability, and cytotoxicity. This system gives quantitative information about the viability, morphology, and number of the cultured cells using electrical impedance.^24^

The aim of this study was to evaluate the cytotoxic or proliferative effect of melatonin and oxyresveratrol on DPSCs for 72 hours. The null hypothesis was that melatonin and oxyresveratrol would have no significant cytotoxic or proliferative effect on DPSCs.

## Materials and Methods

### Cell Culture

Dental pulp stem cells were obtained from the American Type Culture Collection (catalog number: PT-5025, Lonza, Basel, Switzerland). Dental pulp stem cells frozen with 10% dimethyl sulfoxide (DMSO, Sigma-Aldrich, St. Louis, Mo, USA) at −80°C were thawed in a 37°C water bath (Memmert, Schwabach, Germany) and were incubated in a 25 cm^2^ ^2^ tissue culture polystyrene flasks at 37°C in high humidity, 5% CO_2_, 95% air incubator (NuAire^®^, Plymouth, Minn, USA). The culture flask was monitored daily and the culture medium was changed once every 3 days. Cells were passaged on the seventh day when they were approximately 90% confluent. After obtaining background measurements of xCELLigence^®^ device (ACEA Biosciences, Inc., San Diego, Calif, USA), 5000 cells were seeded into 21 wells of an E-Plate^®^ (ACEA Biosciences) containing 100 µL of Dulbecco modified essential medium (DMEM, Lonza, Verviers, Belgium) per well and incubated for 24 hours. Ethics committee approval was received from the Ethics Committee of Atatürk University Faculty of Medicine Clinical Investigations (approval number: 8/28 - 27.12.2018). Informed consent is not applicable, because the study was performed on DPSC derived from the American type culture collection.

### Preparation of Solutions

In a consequence of the dissolution of melatonin and oxyresveratrol in 99.5% DMSO (Sigma-Aldrich, St. Louis, Mo, USA), they were diluted with DMEM, and their stock solutions were obtained at 0.037% and 0.012% concentrations, respectively. Then, initial solutions were diluted with DMEM to obtain 3 different concentrations for melatonin (100 pM, 100 nM, and 100 µM, Sigma-Aldrich) ^25-27^ and oxyresveratrol (10 µM, 25 µM, and 50 µM, Sigma-Aldrich) ^28, 29^ as treatment groups. After the solution in the E-Plate^®^ was removed at the 24th hour, 100 µL of freshly prepared solutions were added to these wells (n = 3). In addition, 100 µL DMEM with DMSO was prepared as the control group.

### xCELLingence Assay

Finally, in order to observe the cytotoxic effect of melatonin and oxyresveratrol on DPSCs, E-Plate^®^ was placed in the xCELLigence^®^ device and incubated. Dental pulp stem cells were monitored every 15 minutes for a period of 72 hours using the xCELLigence^®^ system. As a result of the assay, time-dependent proliferation graphs were obtained using the RTCA-integrated software of the xCELLigence^®^ system (ACEA Biosciences, Inc., San Diego, Calif, USA). The technical procedures were performed by a blinded examiner who had no knowledge of which group was being analyzed.

### Statistical Analysis

The statistical evaluations used in this study were performed with the Statistical Package for the Social Sciences 25.0 (IBM Corp., Armonk, NY, USA) statistical program at a significance level of 5%. By using time as a covariate in the analysis of covariance (ANCOVA) model, the adjusted differences in cell index values between the groups were examined. Furthermore, paired contrast of treatment and time interaction combinations were used to test the differences between the slopes of the regression lines.

## Results

**Figure 1. A, B.**
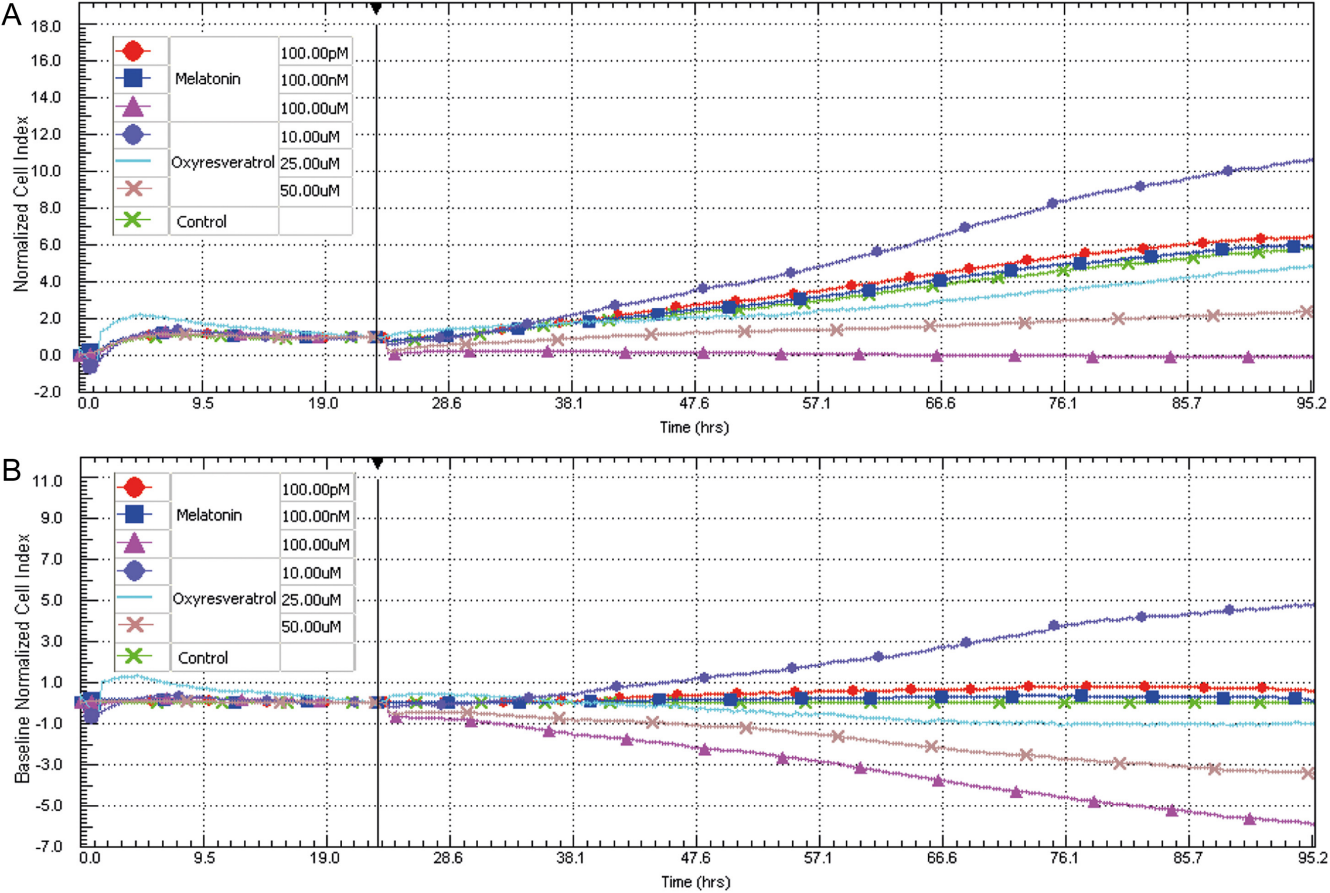
(A) Cell index change and (B) standardized cell index change compared to the control group observed in xCELLigence® for 72 hours following the addition of melatonin and oxyresveratrol to DPSCs at the 24th hour.DPSCs, dental pulp stem cells.

The impedance averages obtained from each group wells were expressed as arbitrary units called cell index. Cell index changes observed in xCELLigence® for 72 hours following the addition of melatonin and oxyresveratrol to DPSCs at the 24th hour is given in Figure 1a. Cell index values in all wells were normalized at the 24th hour using RTCA. A second graph (Figure 1b) using the control group as a reference was prepared to observe cell index changes between groups more clearly.

**Figure 2.**
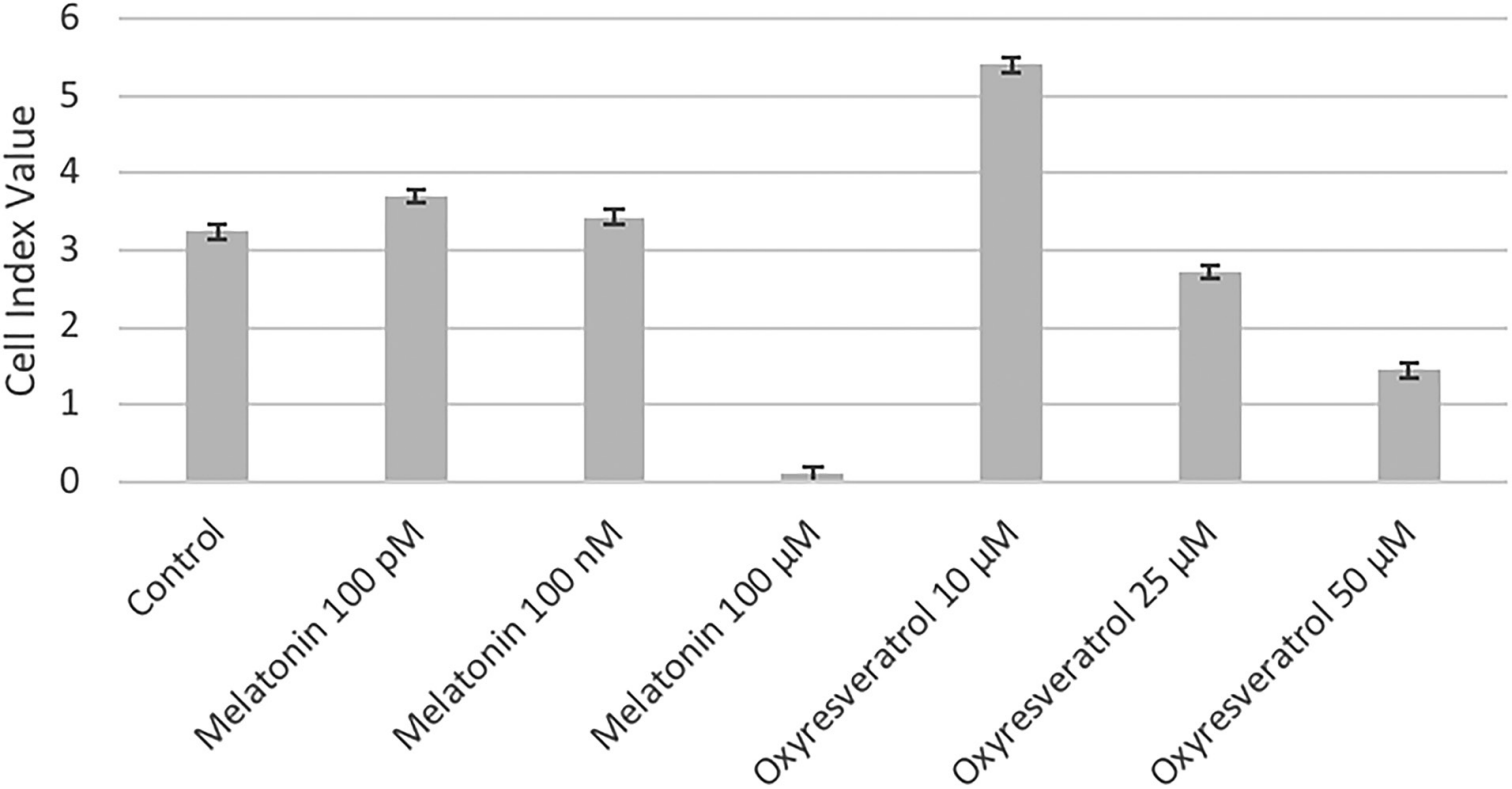
Estimated marginal means with 95% CIs of DPCSs’ cell index valuesas determined by ANCOVA at the 60th hour. All interactions between groups were significant (*P *< .001) except for the difference between melatonin 100 nM and the control group. ANCOVA, analysis of covariance.

Time-dependent differences and differences between the groups were found to be significant (*P *< .001). In order to explore the interaction between the groups and time ANCOVA analysis was used and a significant interaction was observed (*P *< .001). Cell index values of the groups at the 60th hour are given in Figure 2. Paired contrast test showed that all interactions between groups were significant (*P *< .001) except for the difference between melatonin 100 nM and control group (*P *= .126).

Melatonin 100 µM, oxyresveratrol 50 µM, and oxyresveratrol 25 µM groups had lower cell index values (cytotoxic effect) than the control group. While the melatonin 100 nM group had a similar proliferation rate as the control group, the melatonin 100 pM and oxyresveratrol 10 µM groups had higher proliferation rates (Figure 2).

**Figure 3. A, B.**
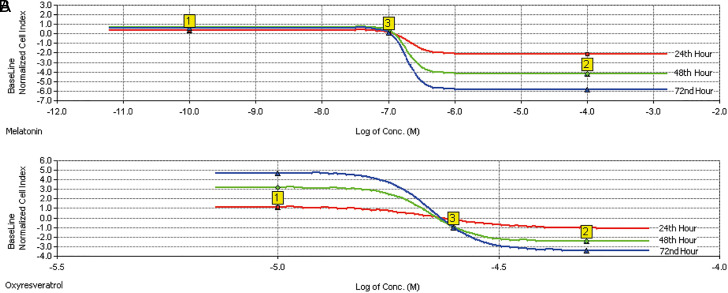
Dose–response curves of A) melatonin and (B) oxyresveratrol at the 24th, 48th, and 72nd hours.

Dose–response curves obtained from cell index values of melatonin and oxyresveratrol at the 24th, 48th, and 72nd hours are presented in Figure 3. From these curves, inhibitor concentration (IC_50_) values, which caused a 50% reduction in cell proliferation, were obtained. IC_50_ values at the 24th, 48^th^, and 72nd hours were 946 nM, 1220 nM, and 1243 nM for melatonin, and 23 µM, 22.2 µM, and 22.5 µM for oxyresveratrol, respectively. In addition, in these time periods, melatonin was found to be 24 times, 18 times, and 18 times more toxic than oxyresveratrol with the same molarity, respectively.

## Discussion

Although the effects of melatonin and oxyresveratrol on ROS have been studied in detail, researches for their effects on DPSCs are limited. Our aim was to compare the proliferative and cytotoxic effects of melatonin and oxyresveratrol at various doses on human DPSCs. According to the results of this study, lower doses of these antioxidant agents increased DPSCs proliferation. 

For the assessment of cell viability and proliferation, single endpoint assays such as XTT, MTT, WST-1, and fluorescence microscopy are preferred. However, the disadvantage of these methods is the difficulty in maintaining the same conditions for all time intervals in measurements. Since the xCELLigence^®^ device used in this study was able to measure in real-time, there was no need for separate cell cultivation for all time intervals to be tested, as in MTT and other conventional test methods.^24^ As standardization can be achieved with this feature, it is possible to obtain easier, reliable, and real-time results in cell culture studies.

In studies evaluating the efficacy of different antioxidants on DPSCs, cell proliferation was found to be increased or unaffected. Ginsenosides, a sub-variant of saponin (exogenous antioxidant), were found to cause a significant increase in cell proliferation on human DPSCs at doses of 0.5-10 µmol/L (especially 5 µmol/L).^30^ Similarly, the lowest dose of NAC increased the number of DPSCs (0.1 mM), while the highest dose (2.0 mM) was found to inhibit cell proliferation compared to the control group.^31^ The studied doses of cinnamaldehyde, obtained from cinnamon did not show proliferative or cytotoxic effects on human DPSCs (1-50 µM).^32^ In our study, melatonin and oxyresveratrol increased DPSCs proliferation, similar to ginsenosides and NAC. They are most similar to NAC as they increase DPSCs proliferation in low doses and show toxicity in high doses.

Due to the positive effects of antioxidant substances on pulp, they were used as direct pulp capping materials in animal studies. In dogs with mechanically exposed class V cavities, the antioxidant enzyme catalase provided better tissue healing than the control group.^33^ When flavonoids, obtained from propolis used as capping material in exposed class I cavities in rats, dentin bridge formation was observed in contrast to zinc oxide eugenol. This was thought to be due to the antioxidant and anti-inflammatory properties of flavonoids.^34^ The determination of proliferative doses of melatonin and oxyresveratrol, known to have antioxidant and anti-inflammatory properties on the DPSCs, may suggest their use as a direct capping material or combination with capping materials in further studies.

Melatonin and oxyresveratrol have successful results on different cell cultures.^25,27,29^ However, since there were limited researches with antioxidants on DPSCs, melatonin, and oxyresveratrol were preferred in this study due to their regenerative-antioxidant properties.

Because of the limited number of studies that measure the efficacy of melatonin and oxyresveratrol on DPSCs, the doses in this study were determined considering DPSCs or other cell culture studies.^25-29^

According to our findings, DPSCs proliferation was increased at the lowest dose of melatonin (100 pM), whereas it was inhibited at the highest dose (100 µM). Similar to melatonin, oxyresveratrol increased cell proliferation at the lowest dose (10 µM) and showed toxic effect at higher doses (25 µM and 50 µM). In rats, acute pulpitis resulted in a decrease in serum melatonin levels, whereas intragastric administration of melatonin reduced acute pulpitis symptoms.^35^ Both results suggested that melatonin might have a therapeutic effect in pulpitis.

In another study, the effects of melatonin at 10^−8^ M, 10^−10^ M, and 10^−12^ M concentrations on DPSCs were evaluated for 5 days by using cell counting kit and it was found that the toxic effect was increased with the elevated dose of melatonin, similar to our study.^26^ However, in contrast to our results, inhibition of DPSCs proliferation was observed on the third, fourth, and fifth days in all melatonin-treated groups. Although similar doses were used in our study, there was a difference between the doses in terms of decreasing and increasing cell proliferation. This variation might be caused by the differences in the study methods. xCELLigence® is able to perform real-time measurement so it can provide more accurate results than other conventional methods. However, further cell culture studies on this subject will provide a more reliable approach.

In an in vitro study evaluating the effect of different resveratrol doses (0.25-100 μM) on mouse fibroblast cells, it was reported that 0.5 μM resveratrol increased cell proliferation at the highest level, while 10 μM, 25 μM, and 50 μM doses decreased cell proliferation compared to the control group.^29^ Although similar doses of oxyresveratrol were used in our study, the use of different cell cultures might have led to different results.

IC_50_ values were finally calculated to confirm the accuracy of the selected doses. Since IC_50_ values were within the studied dose range, it was proved that appropriate doses had been selected at the beginning of our study. Furthermore, the determination of IC_50 _values in our study would provide a reference for dose determination in further studies.

When different concentrations of melatonin and oxyresveratrol were evaluated, maximum DPSCs proliferation was seen in oxyresveratrol in 10 μM group, the most toxic effect was detected in melatonin 100 μM group, and IC_50_ values for oxyresveratrol were observed to be higher than melatonin. Based on these results, oxyresveratrol was thought to be more successful than melatonin in DPSCs proliferation rates. As limited studies on this subject were found in the literature, further studies in this field are recommended.
